# Power Flow in a Large-Core Multimode Fiber under External Perturbation and its Applications

**DOI:** 10.1038/s41598-017-01117-4

**Published:** 2017-04-19

**Authors:** Sen Qian, Yang Xu, Lisheng Zhong, Lei Su

**Affiliations:** 1 State Key Laboratory of Electrical Insulation and Power Equipment, Xi’an Jiaotong University, Xi’an, China; 2 School of Engineering and Materials Science, Queen Mary University of London, London, UK

## Abstract

Large core optical multimode fiber provides benefits such as a large light-coupling tolerance, easy handling, and delivery of higher light power without undesirable nonlinear effects. In this research, we exploit the effects of external perturbation on the power flow within the large core fiber and present two relevant applications, namely a perturbation sensor and a doughnut beam tuner. Since conventional multimode fiber power flow model does not take into consideration the perturbation effect, we modify the power flow model so that the influence of time varying perturbation can be theoretically analyzed. Based on our theory, we further conduct the numerical simulation and experiments on these two applications. For the fiber vibration sensor, the proposed numerical model shows that the sensor sensitivity depends on the intensity profile of the launched beam and also the higher-order harmonics that were not reported previously can become interferences to affect the signal. For the beam tuner application, we prove both theoretically and experimentally that the doughnut intensity profile at the fiber output can be tuned in real-time by applying external perturbations to the fiber. We expect that the results can be useful to further exploit the external perturbation on large core fiber in various applications.

## Introduction

Large core multimode fiber (MMF) offers the benefits such as a large light-coupling tolerance, easy installation and handling, convenient integration with low cost optoelectronics, and efficient delivery of high power light without inducing strong nonlinear effects^1, 2^. The mode coupling in large core multimode fiber has attracted much interest, as it determines a variety of important parameters such as the transmission bandwidth and the loss in fiber communication systems^3, 4^. However, because of the excessive number of modes involved, it is challenging to obtain the exact solutions of all the modes within the large core fiber to implement the field coupling model or power coupling model^5^ for mode coupling research. Power-flow model, as an approximation approach, was developed to study the mode coupling within a large core multimode fiber and has found many applications in optical fiber communications^2–4, 6–8^. However, previous research paid little attention to the influence of external perturbations on the power flow among modes in a multimode fiber, which should be an important consideration.

In this work, we investigate the effects of external perturbations on the power flow within a large core optical fiber. We first improve the conventional power flow model to incorporate a term that reflects the external perturbation effect. Then, through simulations and experiments, we exploit two applications of large core multimode fibers under perturbations, namely a vibration sensor and a doughnut beam tuner. For the vibration sensor, the external perturbation changes the phase modulation and mode coupling within the fiber and this leads to the change at the sensor output. Our improved model provides a new numerical solution to large core fiber sensor design and identifies a few highly influential factors such as sensor’s sensitivity. Such factors, however, were neglected in previous research^9–12^. The second application we present by using our model is a large-core multimode fiber doughnut beam tuner. Doughnut beams are now widely used in communication^13, 14^, microscopy^15, 16^, laser drilling^17^, optical trapping^18^, and laser surgery^19^. The intensity profile of the doughnut beam needs to be adjusted to achieve specific purposes. One example is the stimulated emission depletion microscopy for super resolution imaging^20^, in which the doughnut intensity profile influences the resolution. As another example, in laser surgery and laser drilling, the intensity profile of the doughnut beam is required to realize desirable heating or drilling patterns^17, 21^. Additionally, doughnut beam was used in optical trapping as a sperm sifter^22^. Previous research demonstrated that the doughnut beam can be generated by hologram, spatial light modulator, diffractive optics, metal plate, and multimode fiber interference^23–27^. In this paper, based on simulations and experiments, we show that the external perturbations can make a large core multimode fiber an easy-to-use and compact doughnut beam tuner.

## Results

### Modified Power Flow Equation

Our modified power-flow equation is shown as Equation (1) below. The mathematical derivation is given in the Methods Section.

1 $$\frac{\partial P(\theta ,z,t)}{\partial z}=\frac{{D}_{0}{(1+b(t))}^{2}}{\theta }\frac{\partial }{\partial \theta }(\theta \frac{\partial P(\theta ,z,t)}{\partial \theta })$$

where, *P*(*θ*, *z*, *t*): the angular power distribution; *z*: propagation distance; *θ*: propagation angle with respect to the core axis; *t*: time; *D*_*0*_: static coupling coefficient; *b*(*t*): induced coupling coefficient.

The main difference between our modified power-flow equation and conventional power-flow equation (Equation (5) in Methods) lies in the introduction of the time varying term *b*(*t*). Because Equation (1) involves the time variable *t*, a new algorithm different from the conventional one is needed to solve the equation. However, we expect that the algorithm used for the conventional power flow equation is still valid, provided that the time duration for the light travelling through the MMF is much shorter than the varying period of the perturbation. For example, for a perturbed fiber of 10 m, it takes only 50 ns (corresponding to a frequency of 20 MHz) for the light to travel through. Hence, if the varying frequency of the perturbation is much lower than 20 MHz, the time-varying coupling coefficient can be regarded as quasi-static. Therefore, Equation (1) can be numerically solved by substituting the induced coupling coefficient *b*(t) into Equation (1) first and then drop the time variable to simulate it based on the algorithm designed for conventional power flow equation.

### Large core MMF as a perturbation sensor

We first used Crank Nicolson scheme to numerically simulate the power flow distribution within large core fiber under external perturbations based on Equation (1) that we formulated (detail in Methods), since Crank Nicolson scheme has the advantage of being unconditionally stable and non-dissipative^28^. A Gaussian beam was launched into the large core fiber, which had the expression as,

2 $$P(\theta ,0)=exp(-\frac{{(\theta -{\mu }_{c})}^{2}}{2{\sigma }^{2}})$$

with 0 < θ < *θ*_c_ (*θ*_c_ being the critical angle), *μ*_c_ being the tilt angle, and σ being the standard deviation of power distribution. The critical angle *θ*_c_ was set as 23°, equivalent to a numerical aperture (NA) of 0.39. It is well known that 99% power is constrained in the range [−3σ, 3σ] for a Gaussian beam. Hence σ was set as 1.9° to simulate an input wave from a 0.10 NA single mode fiber (SMF). The tilt angle μ_c_ was set as 0°. The coefficient *D*_0_ was found to vary between 10^−6^ rad^2^/m for silica optic fiber and 10^−3^ rad^2^/m for polymer optic fiber^2–4, 6–8^. Based on a priori estimation on the coupling coefficient of the fiber that was used in this research, we chose 10^−5^ rad^2^/m in our simulation program. The dynamic coupling coefficient in Equation (1) was set as *b*(*t*) = *A*_m_sin (2π*t/T*) to simulate a perturbation of sine wave on a large core fiber, where *A*_m_ represents the modulation amplitude of induced coupling coefficient and *T* is the period of the external perturbation. It was experimentally found that the applied strain could enhance the mode coupling coefficient to two orders of magnitude higher^29^. Therefore, we expect that *A*_m_ can range between [0, 9]. Clearly, there is no induced time varying coupling coefficient when *A*_m_ = 0. However, the coupling coefficient increases to two orders of magnitude higher when *A*_m_ = 9. In our simulation, we used *A*_m_ = 1 and *A*_m_ = 2, and the propagation distance was 4 m.

**Figure 1:**
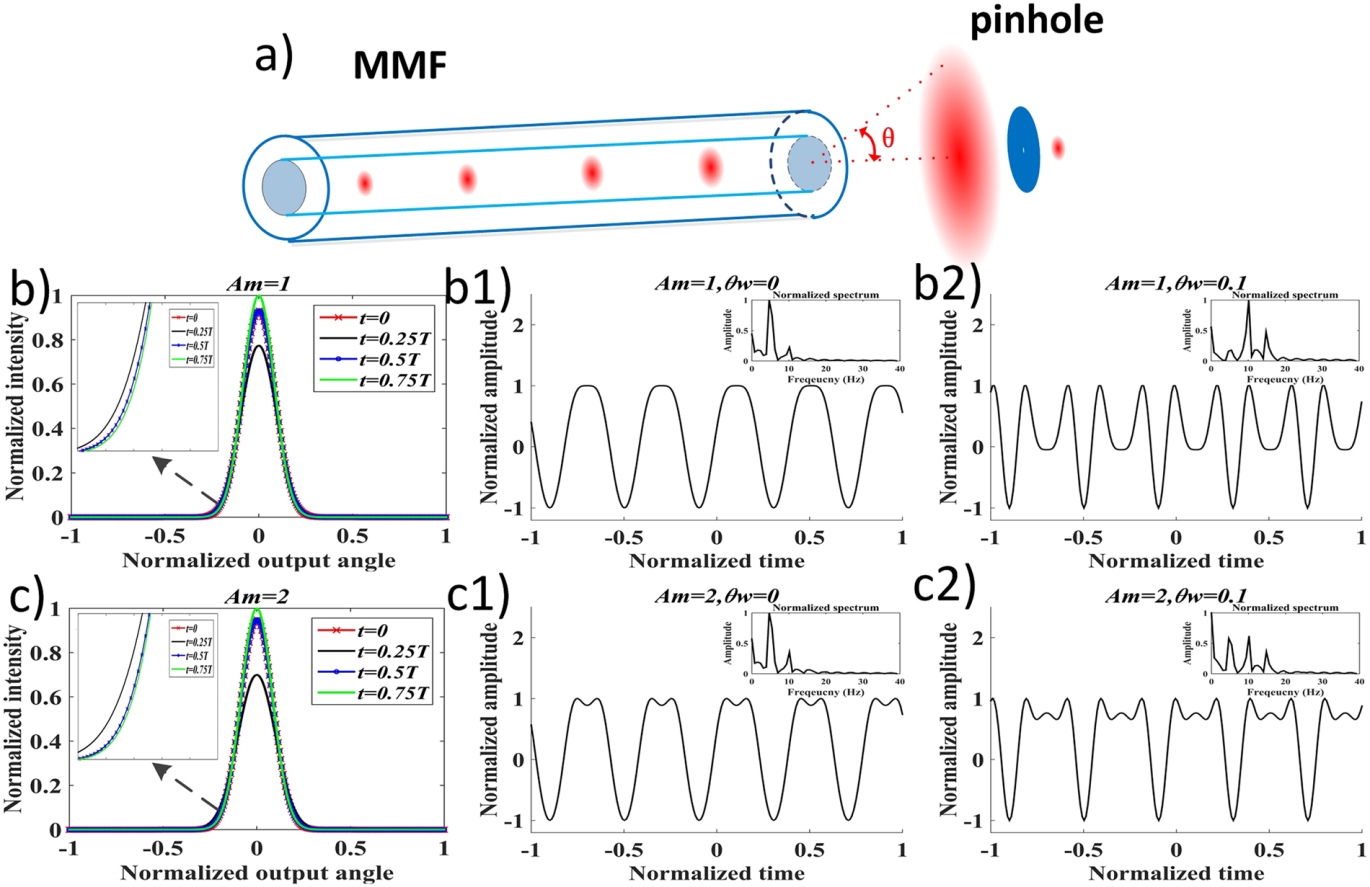
Numerical simulation results of the angular power flow distribution of a large core MMF under external perturbations. (**a**) shows the schematic illustration of power coupling within the MMF among different principal mode groups at different propagation angles. External perturbations can tune the mode coupling and hence the power flow. The power flow that passes through a pinhole can manifest the perturbation signal pattern. (**b** and **c**) show the angular intensity profiles from MMF when the coupling coefficient in Equation (1) was *D* = *D*_0_(1 + *A*_m_sin(2π*t*/*T*))^2^, where *T* was the period of external perturbation, *D*_0_ was the static coupling coefficient and *A*_m_ was the modulation amplitude of induced coupling coefficient by the perturbation. A selecting window (pinhole) of size 0.01 (normalized unit) that only allowed part of light to be detected was placed with its center *θ*_w_ at 0 and 0.1, which gave rise to the signal patterns (**b1**) and (**b2**) for *A*_m_ = 1 and signal patterns (**c1** and **c2**) for *A*_m_ = 2. The insets in (**b** and **c**) illustrate that stronger mode coupling lead to optical power flow from smaller angle modes into larger angle modes. However, the total power was conserved since there was only power exchange among the guided modes, i.e. the areas under different intensity profiles in (**b** and **c**) are same. The insets in (**b1**,**b2**,**c1** and **c2**) are the frequency spectra corresponding to the temporal waveforms.

The normalized angular intensity profiles of the power flow output from the large core fiber under perturbation when *t* was 0, 0.25*T*, 0.5*T* and 0.75*T* are shown in Fig. 1. Clearly, the intensity profile at the fiber output varied when the induced coupling coefficient changed. The power carried in the smaller angle modes flowed into the larger angle modes. As the modulation amplitude of the induced coupling coefficient increased, the intensity profile variation became stronger. This can be seen that intensity profiles in Fig. 1(c) differ more than the ones in Fig. 1(b). A sensor to detect the perturbation can be configured by placing a filtering window (generally a pinhole) at the fiber output image plane to only detect a portion of the power flow output. In our simulation, we used a detection window of 0.25°, corresponding to 0.0109 in the normalized unit of a fiber with a critical angle of 23° (detailed in Methods). The window was placed at 0° and 2.5°, corresponding to 0 and 0.109 in the normalized unit, to detect the power flow variation covered by this window, as shown in Fig. 1(b1,b2) for *A*_m_ = 1 and Fig. 1(c1,c2) for *A*_m_ = 2. The time domain contained 5 periods, i.e. [0, 5*T*]. The result suggested that the intensity profile variation within the filtering window gave rise to a signal of 5 periods in all the signal patterns in our simulation. Moreover, the signal exhibited different patterns where the harmonics were also present in the temporal waveforms. Frequency spectra are included as insets to show the harmonic components. The 2^nd^ harmonic, 10 Hz component, can be seen in the spectra. This effect was explained by a descriptive model^9, 30^ previously. However, our numerical result further demonstrates that the 3^rd^ harmonic may be present, as shown in (b2) and (c2).

**Figure 2:**
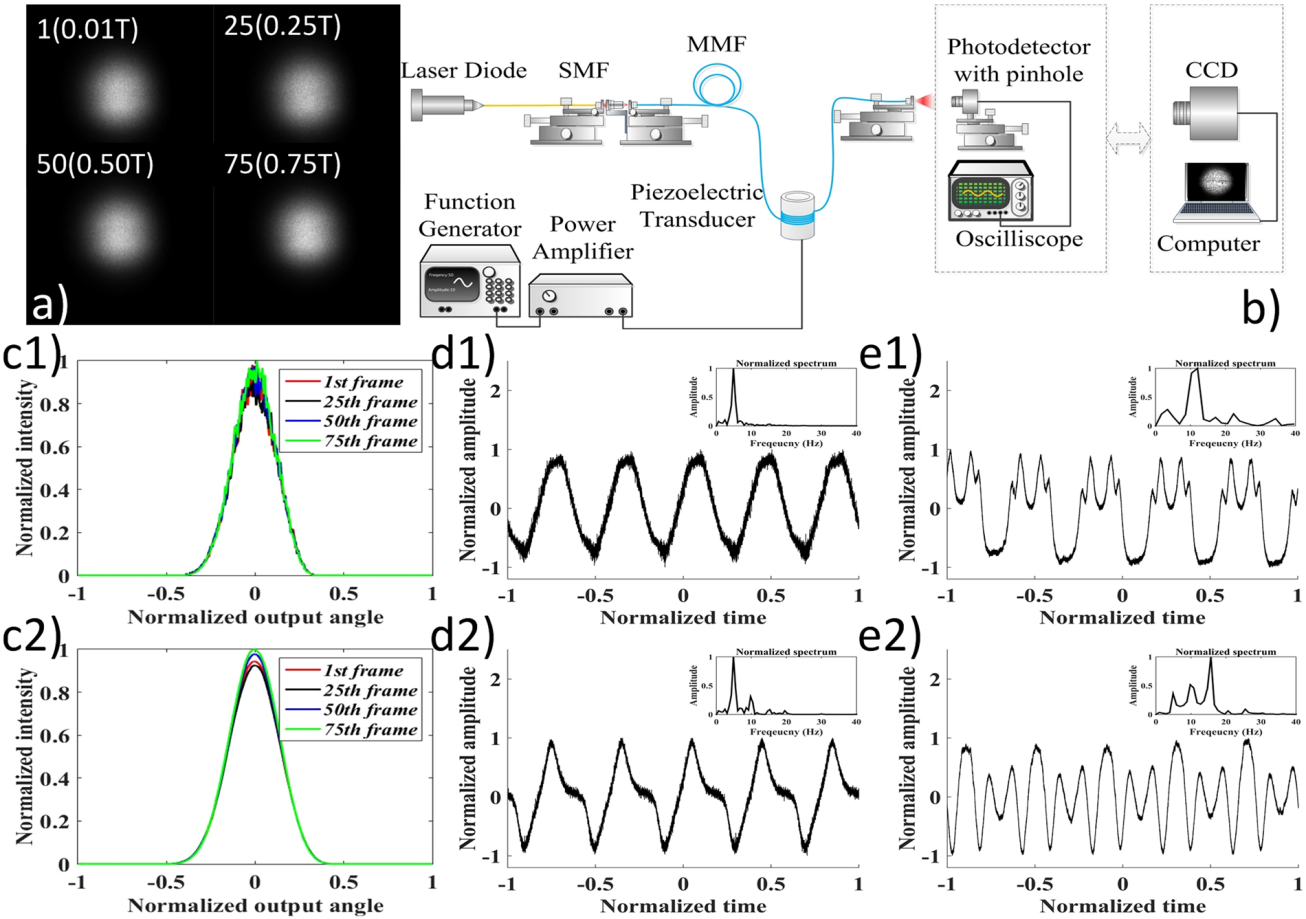
Experimental results of the angular power distribution of a large core multimode fiber under external perturbations. (**a**) shows the output images of 1^st^, 25^th^, 50^th^, 75^th^ frames acquired by the CCD camera. 100 frames corresponded to a period (T) of perturbation. (**b**) shows the schematic experimental layout. (**c1**) shows the intensity profile along the 512^th^ middle row of these four frames in (**a**). (**c2**) shows the corresponding intensity profiles after smoothing the profiles in (**c1**) in order to eliminate the sawtooth. The result indicated that the power coupling due to external perturbations is more efficient at the output center. (**d1** and **d2**) show two captured 5 Hz signal by photodetector module. (**e1** and **e2**) show two captured signals when drive voltage was increased to 100 Vpp. The 5 Hz and harmonics (10 Hz, and 15 Hz) signals were detected. The insets in (**d1**, **e1**,**d2** and **e2**) are the frequency spectra corresponding to the temporal waveforms.

We setup an experiment to study the power flow within a large core fiber sensor under external perturbations. The schematic diagram of the experimental setup is shown in Fig. 2(b). The output from a SMF-pigtailed 642 nm laser diode was collimated and focused by a 0.10 NA objective into the MMF. The multimode fiber has an optical loss figure of 2 dB/km. Hence the total losses of the 4 m fiber used in the vibration sensor and the 40 m fiber involved in the doughnut beam tuner were less than 0.1 dB, which was negligible. The output power from the SMF pigtail was adjusted to 8 mW. The MMF had a 200 μm core diameter and a 0.39 NA. We wrapped 50 turns of the MMF, approximately 4 m in total length, around a piezoelectric transducer (PZT). The drive voltage was generated by a function generator, and was subsequently amplified by a power amplifier and applied to the piezoelectric transducer to simulate the vibration. Two detection modules were used, including a photodetector with a 1 mm pinhole and a high speed 1024 × 1280 pixel CCD camera.

We first actuated the PZT by a 5 Hz 40Vpp sine wave, and imaged the intensity profile of the power flow outputted from the MMF. The frame rate of the CCD camera was set as 500 Hz, and therefore 100 captured frames corresponded to a period of the 5 Hz signal. The output of the 1^st^, 25^th^, 50^th^, and 75^th^ frames are shown in (a) and their intensity profiles along the 512^th^ row are shown in Fig. 2(c1). Because of the sawtooth feature along the intensity profile, we used an average filter of 32 points to smooth the captured intensity profiles to show their differences, as shown in Fig. 2(c2). Clearly, the experimental intensity profiles resembled the simulation results that the power exchange primarily occurred at the output center. Then we replaced the CCD camera module with the photodetector module to observe the local power flow variation. The temporal waveforms are shown in (d1) and (d2), with the insets showing their corresponding frequency spectra. The 5 Hz was dominant in the waveform, whereas a weak 2^nd^ harmonic, the 10 Hz component, was present in (d2). When the applied voltage was further increased to 100 Vpp, the 10 Hz component was increased, as shown in both (e1) and (e2). The 15 Hz component was also present in (e2). In this case, assuming a 5 Hz and a 15 Hz signals were applied on the large core fiber sensor simultaneously, the third-order harmonics of the 5 Hz signal would overlap with the fundamental-frequency signal of 15 Hz signal. Consequently, these two frequency components cannot be distinguished.

**Figure 3:**
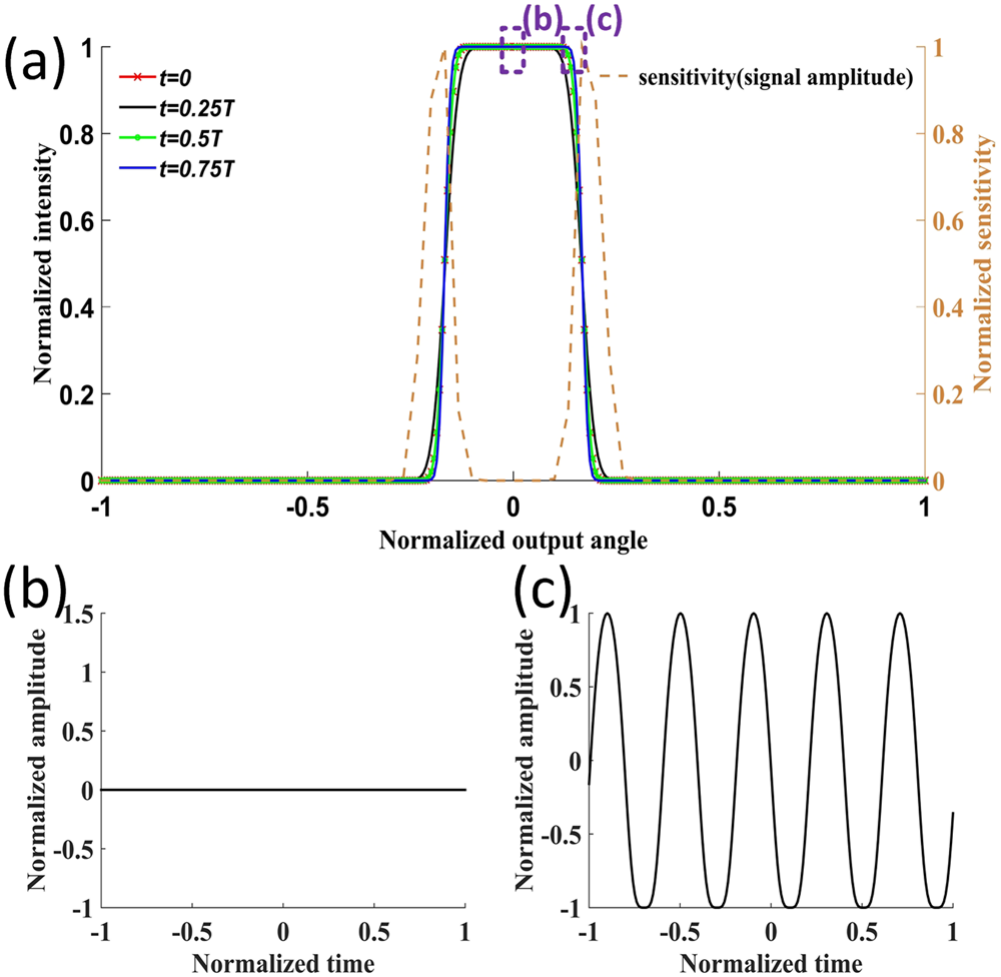
Simulation results of the sensitivity dependence of a large core fiber vibration sensor on the intensity profile when a top hat intensity profile was launched at the input. (**a**) shows the angular intensity profile at the MMF output when the coupling coefficient in Equation (1) was *D* = *D*_0_(1 + *A*_m_sin(2π*t*/*T*))^2^ at 0, 0.25*T*, 0.50*T*, 0.75*T*. A 0.01 size window was used to scan the angular distribution and to obtain the signal response. The amplitude of the signal response was used as the sensitivity, shown as the brown dashed curve. Two representative signal patterns detected by the windows are given in (**b**) at the center of the output and (**c**) at the edge of the intensity cliff. The difference between Fig. 3 and Fig. 1 is that different intensity profiles were launched at the fiber input, i.e. top hat in Fig. 3 and Gaussian in Fig. 1. A comparison between Fig. 3 and Fig. 1 suggests that the sensitivity depends on the intensity profile. For the top hat intensity profile, the sensitivity was highest at the edge of the intensity cliff as shown by the brown dashed curve. However for the Gaussian profile, the sensitivity was highest at the output center as illustrated in Fig. 1.

The modified power flow equation can also be used to study the relationship between the sensitivity of the large core fiber sensor and the intensity profile. In contrast to the Gaussian profile, a top flat beam was launched to the large core fiber. The dynamic coupling coefficient was still set as *b*(*t*) = *A*_m_sin (2π*t/T*). The propagation distance was 4 m. The intensity profiles are shown in Fig. 3(a) when *t* was 0, 0.25*T*, 0.5*T* and 0.75*T*.

In the top hat intensity profile shown in Fig. 3, the intensity abruptly drops at the edge of the intensity cliff. This means that for a top flat intensity profile, the power exchange due to the perturbation is most efficient at the edge of the intensity cliff. To clearly demonstrate this, we used a 0.01 size window to scan across the intensity profile to detect the power variation under external perturbations. Two representative detected signal patterns are shown in Fig. 3(b and c), where the filtering window was placed at the center and at the intensity cliff, respectively. No signal variation was detected when the window was centrally placed. However, when the window was placed at the intensity cliff, a signal corresponding to external perturbation was detected. The definition of the sensitivity of a sensor is the output signal amplitude in response to the input excitation. The sensitivity profile is shown in Fig. 3(a) as the brown dashed curve, which matches to the above discussions very well. Previously, we used a simplified descriptive model to explain this dependency of the sensitivity^10^. The result presented here, in contrast to previous research^9–12^, provides a modified power-flow model that incorporates the effect of external perturbations, which can be used as a guide for future optical speckle sensor design.

### Large core MMF as doughnut beam tuner

**Figure 4:**
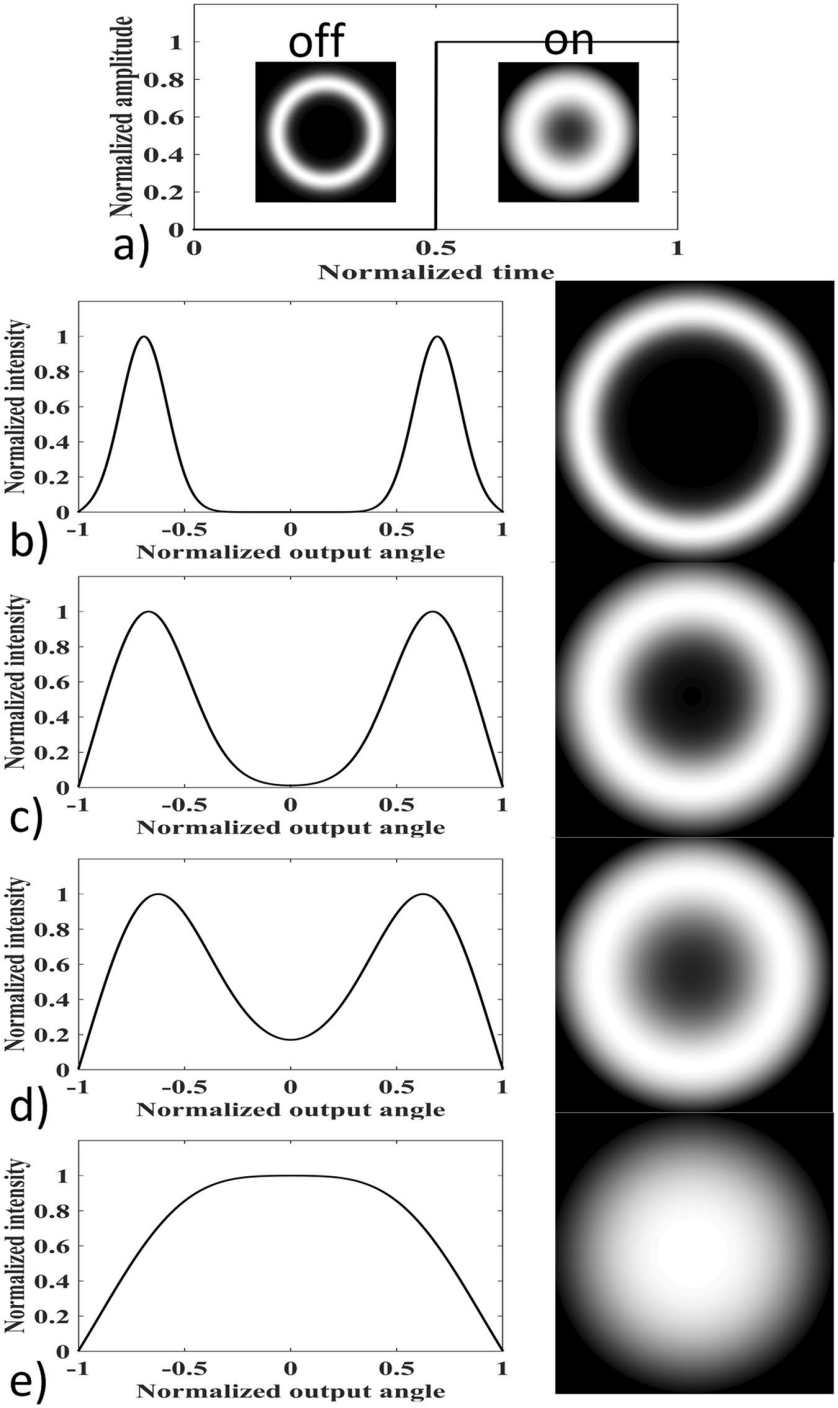
Simulation results of tuning doughnut beam profiles through perturbations applied on a large core MMF. (**a**) shows the schematic diagram of the simulation for doughnut profile tuning using square-wave perturbation. During perturbation On, more power flowed into the center of the doughnut. (**b**,**c**,**d** and **e**) show the doughnut and its associated intensity profiles when different amplitudes of perturbations were introduced. (**b**) *A*_m_ = 0 (perturbation Off), (**c**) *A*_m_ = 1.5, (**d**) *A*_m_ = 2.5, (**e**) *A*_m_ = 6.

We also envisaged that by applying external perturbations to a large core fiber it can function as a dynamic doughnut beam tuner. We first used the simulation to investigate tuning a doughnut beam through perturbation effects on the large core fiber, and the results are shown in Fig. 4.

In our simulation, a square wave was applied to the large core MMF as the external perturbation to change the mode coupling. As shown in Fig. 4(a), the square wave had the form of *D*_0_(1 + *A*_m_ *ε*(t))^2^, where *ε*(t) was a step pulse, and imposed a 50% duty cycle perturbation on the fiber. The fiber length was 40 m and *D*_0_ was 10^−5^ rad^2^/m. The corresponding doughnut profiles were changed during the perturbation “On” and “Off”. Figure 4(b) showed the doughnut profile during perturbation Off, i.e. A_m_ = 0. (c), (d) and (e) show the doughnut profiles during perturbation On when A_m_ = 1.5, 2.5 and 6.

The results demonstrated that the doughnut profile can be tuned by external perturbations, and different perturbation amplitudes can further induced different degrees of mode coupling in the fiber. Under strong perturbations, i.e. strong mode coupling, the doughnut profile can be converted to a super Gaussian profile with a relatively flat top.

**Figure 5:**
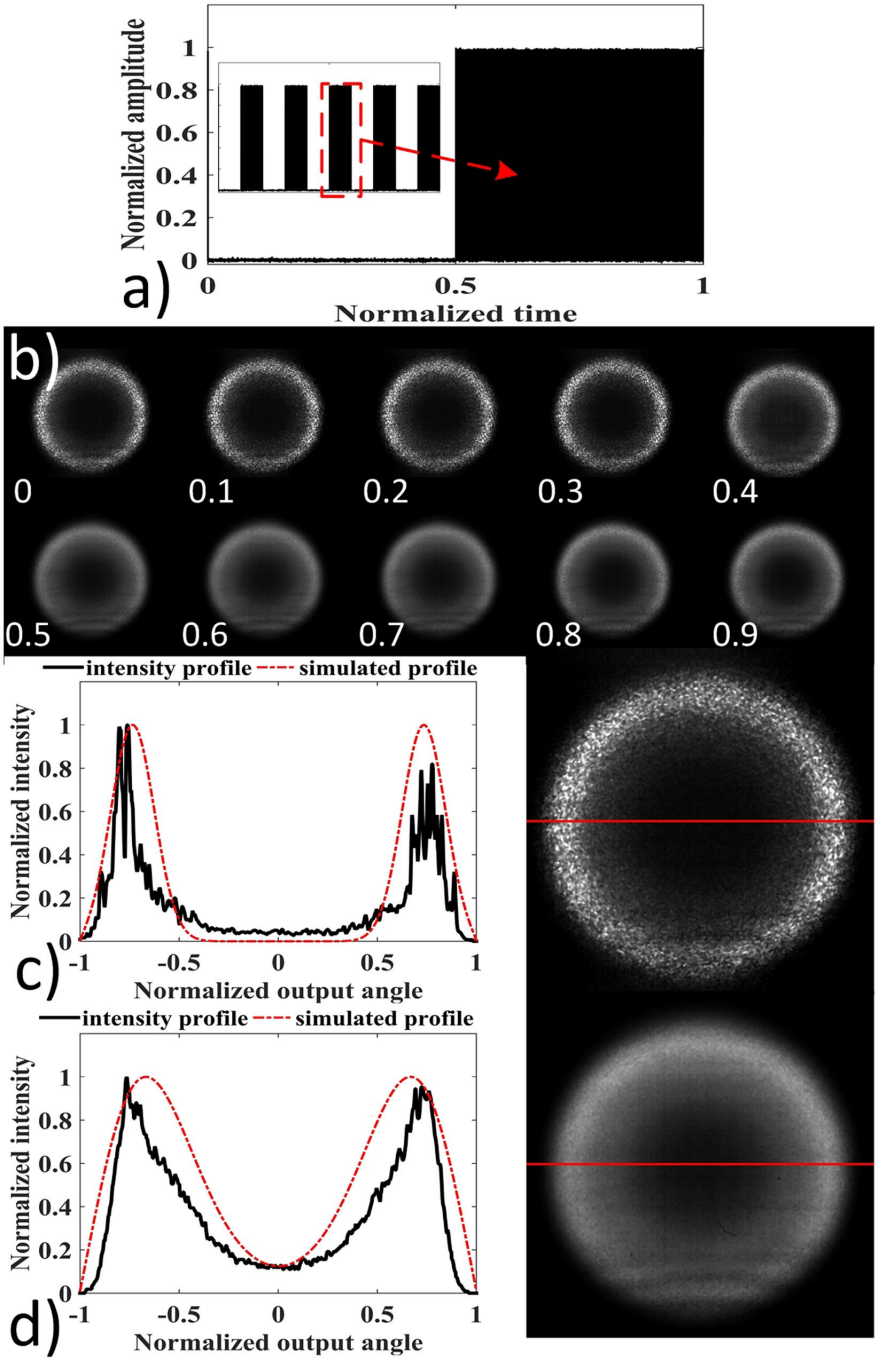
Experimental results of doughnut beam profile tuning through external perturbations. (**a**) 50 Hz and 50% duty cycle square pulse actuated PZT which emitted ultrasonic pulse to perturb the fiber. A series of images captured by the CCD camera operated at 500 Hz during 20 ms corresponding to a period of perturbation are also shown in (**b**). (**c** and **d**) show the 1^st^ and 6^th^ doughnut (0.1 and 0.6), and the intensity profile along the middle row (512^th^ shown as red line) were also analyzed. The simulated profiles in Fig. 4(b and d) are also shown as red dashed curve.

We also setup a proof of concept experiment to test the proposed MMF doughnut beam tuner. The experimental layout was similar to the one in Fig. 2(b). The SMF fiber pigtail from the laser was tilted at 16° to launch the beam into the MMF to only excite larger angle modes. A PZT module was used to introduce mode coupling to the 40 m multimode fiber. When the power of the module was turned on, the square wave consisting of high frequency sinusoidal waves with 50 Hz repetition rate was applied onto the PZT to perturb the fiber as shown in Fig. 5(a). The CCD camera was operated at 500 Hz, and the images captured during 20 ms corresponded to a period of the perturbation. Figure 5(b) shows the captured sequences in a period. The result clearly proved experimentally that the external perturbation can tune the doughnut profile. To illustrate this, the intensity profile along the 512^th^ middle row of the 1^st^ and 6^th^ images (0.1 and 0.6), red lines in (c) and (d), were analyzed. In comparison to (c), more optical power flowed into the center of the doughnut in (d) because of the extra mode coupling induced by external perturbation. Another noticeable change is the smoothness of the curve. It can be seen that the perturbation applied to the large core MMF also reduced the speckle contrast, as the sawtooth along the intensity curve was smoothed in (d) in comparison to (c). This effect was previously discovered and has been used in imaging applications^30^. In summary, the proof of concept experiment proved a dynamic doughnut beam tuner using a large core multimode fiber through external perturbations.

## Discussion

In this paper, we studied the effect of external perturbations on large core multimode fibers and its applications. An improved power-flow equation that incorporates the perturbation effect on large core fiber was developed based on conventional power flow equation. The results demonstrate that the perturbation signal applied to the large-core multimode fiber can be measured by monitoring the power flow inside the fiber. However, as shown in our results, such power-flow signals may contain higher order harmonics of the external perturbation. This may limit its use in applications where the external perturbation contains a complicated frequency spectrum that is difficult to recover. It is possible to remove the harmonics by introducing additional filters. Another application of large core multimode fiber under perturbation is a doughnut beam tuner. The results illustrate that the doughnut beam profile can be tuned by power flow exchange within the large core multimode fiber induced by external perturbation.

## Methods

The power flow equation was initially derived based on the mode coupling equation between different discrete modes, shown in Equation (3)^6, 9, 30, 31^,

3 $${P}_{m}(z+{\rm{\Delta }}z)-{P}_{m}(z)=-{\alpha }_{m}{P}_{m}(z)+\sum _{n=1 \& n\ne m}^{N}{d}_{mn}[{P}_{m}(z)-{P}_{n}(z)]$$

where *d*_mn_: the mode coupling coefficient between *m*th mode and *n*th mode; *z*: propagation distance; *α*_*m*_: attenuation factor of *m*th mode; *P*_m_(*z*): the power of *m*th mode; *N*: the total number of guided modes.

The mode coupling Equation (3) describes the mode coupling between discrete modes. When the fiber supports a large volume of modes, the discrete mode index number *m* can be replaced by the propagation angle *θ*. Based on the fact that mode coupling mainly dominates between neighboring modes, the following power flow equation was derived^2–4, 6–8^,

4 $$\frac{\partial P(\theta ,z)}{\partial z}=-\alpha (\theta )P(\theta ,z)+\frac{{\delta }^{2}}{\theta }\frac{\partial }{\partial \theta }(\theta d(\theta )\frac{\partial P(\theta ,z)}{\partial \theta })$$

where *P*(*θ*, *z*): the angular power distribution; *z*: propagation distance; *θ*: propagation angle with respect to the core axis; *α*(*θ*): attenuation coefficient; *d*(*θ*): coupling coefficient; *δ*: a constant related to the core radius and light wavelength, given as *λ*/4*an*, where *λ* is the wavelength, *a* is the core radius and *n* is the core refractive index.

**Figure 6:**
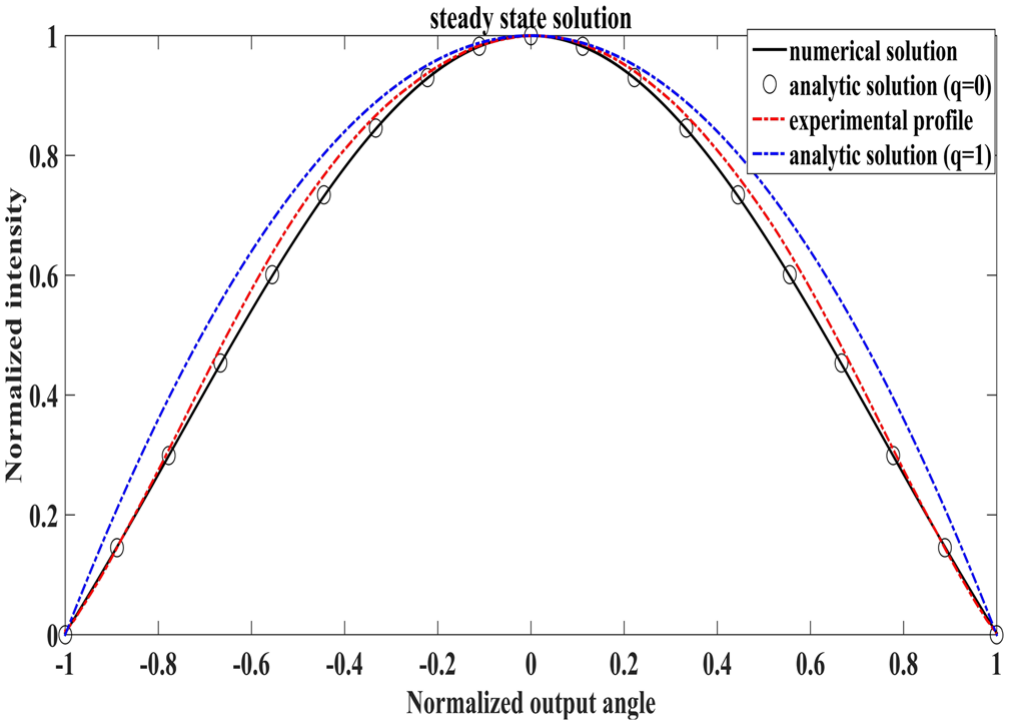
Numerical, analytic and experimental beam profiles under the steady state solution. *q* is the angle dependence order of *d*(*θ*). Experimental profile lies between the theoretical profiles when *q* = 0 and *q* = 1. *q* = 0 was chosen for numerical simplicity in our simulation.

It should be noted that if the launching beam was in impulse waveform, there should be another term composed of time derivative of power flow, ∂*P*/∂*t*, in Equation (4)^32, 33^. However, it is neglected here because the launching beam considered in this research was a CW beam. Equation (4) is an effective method to model the power distribution within a large core MMF subject to mode coupling. The attenuation coefficient, *α*(*θ*), in Equation (3) is caused by scattering and absorption. The attenuation factor was found to be uniform among all the modes except for the higher order ones near the cutoff angle, which can become radiation modes. Therefore, it is reasonable to assume a uniform attenuation for all guided modes that are away from the cutoff angle, which can be neglected when mode coupling is under research interest. The mode coupling coefficient *d*(*θ*) can be regarded as an angle dependent function, given as *d*_0_(*θ*_c_/|*θ*|)^2*q*^, where *d*_0_ is a constant and *q* is the angle dependence order^33, 34^. When *q* = 0, *d*(*θ*) is independent of *θ*. Generally, when the input angular range is small compared to the receiving angle of the multimode fiber (i.e. the NA), *q* can be approximated to 0. The value of *q* can also be determined by comparing the experimental beam profile under steady state with the theoretical steady state beam profiles calculated at different *q*. As shown in Fig. 6, our experimental profile lies between the theoretical profiles of *q* = 0 and *q* = 1. For simplicity, we chose *q* = 0 and therefore *d*(*θ*) = *d*. In this case, Equation (4) can be expressed as,

5 $$\frac{\partial P(\theta ,z)}{\partial z}=\frac{D}{\theta }\frac{\partial }{\partial \theta }(\theta \frac{\partial P(\theta ,z)}{\partial \theta })$$

where *D* = *δ* ^2^ *d* is called the normalized coupling coefficient. The boundary conditions are^2–4^,

6 $$P({\theta }_{c},z)=0;{D\frac{\partial P}{\partial \theta }| }_{\theta =0}=0$$

The first condition means that the power is close to zero at the boundary, and the second condition means that the coupling is limited to the guided modes. It should be noted that the fiber loss is higher for the higher modes since the guided modes can flow into the cladding and become radiation modes or leaky modes. As a result, the angular dependence of the mode loss needs to be accounted if the modes of interest propagate near the cutoff region.

In order to incorporate the time varying perturbation into power flow equation, an expression that reflects the relationship between the normalized coupling coefficient *D* and the external perturbation should be derived. To do this, we first express the coupling coefficient *d*_mn_ as a function of the external perturbation. The perturbation effect changes the mode coupling coefficient *d*_mn_ in Equation (3), and *d*_mn_ can be expressed as^9, 30, 31^,

7 $${d}_{mn}={K}_{mn}^{2}F{({\beta }_{n}-{\beta }_{m})}^{2}$$

where *K*_*mn*_ is a constant depending on the specific fiber, *β*_m_ and *β*_n_ are the propagation constants of *m*th and *n*th modes and *F(β*_*m*_ − *β*_*n*_) is^9, 30, 31^,

8 $$F({\beta }_{m}-{\beta }_{n})={C}_{0}+\frac{1}{\sqrt{{\rm{\Delta }}z}}{\int }_{-\frac{{\rm{\Delta }}z}{2}}^{\frac{{\rm{\Delta }}z}{2}}f(z,t){e}^{-i({\beta }_{m}-{\beta }_{n})z}dz$$

where *f*(*z*, *t*) is called the deformation function, Δz is the length of the fiber under perturbation, and *C*_0_ is the inherent mode coupling coefficient due to the residual strain in the fiber. Since most deformation can be analyzed by Fourier analysis, the trigonometric function is considered, where *f*(*z*, *t*) is *a*(*t*)cos (2π*Nz*/Δ*z*) and *a*(*t*) describes the time varying deformation amplitude and cos (2π*Nz*/*Δz*) describes its spatial distribution^9, 30, 31^, in which *N* is the period of deformation along the *z* axis. Then, Equation (8) can be expressed as,

9 $$\begin{array}{rcl}F({\beta }_{m}-{\beta }_{n}) & = & {C}_{0}+\frac{1}{\sqrt{{\rm{\Delta }}z}}{\int }_{-\frac{{\rm{\Delta }}z}{2}}^{\frac{{\rm{\Delta }}z}{2}}a(t)cos\,(2\pi Nz / {\rm{\Delta }}z){e}^{-i({\beta }_{m}-{\beta }_{n})z}dz\\  & = & {C}_{0}+\frac{a(t)}{\sqrt{{\rm{\Delta }}z}}[\frac{sin([\frac{2\pi N}{{\rm{\Delta }}z}-{\rm{\Delta }}{\beta }_{mn}]\frac{{\rm{\Delta }}z}{2})}{\frac{2\pi N}{{\rm{\Delta }}z}-{\rm{\Delta }}{\beta }_{mn}}+\frac{sin([\frac{2\pi N}{{\rm{\Delta }}z}+{\rm{\Delta }}{\beta }_{mn}]\frac{{\rm{\Delta }}z}{2})}{\frac{2\pi N}{{\rm{\Delta }}z}+{\rm{\Delta }}{\beta }_{mn}}]\end{array}$$

If the fiber vibrates spatially uniformly along the z axis transversally, i.e. *N* = 0, Equation (9) can be simplified as Equation (10),

10 $$F({\beta }_{m}-{\beta }_{n})={C}_{0}+\frac{2a(t)}{\sqrt{{\rm{\Delta }}z}}\frac{sin({\rm{\Delta }}{\beta }_{mn}\frac{{\rm{\Delta }}z}{2})}{{\rm{\Delta }}{\beta }_{mn}}$$

By substituting Equation (10) into Equation (7), assuming *d*_mn_, is quasi uniform based on the aforementioned reason and adding a constant that represents the minimum coupling coefficient, we have,

11 $${d}_{mn}={K}^{2}{C}_{0}^{2}{(1+\frac{\sqrt{{\rm{\Delta }}z}}{{C}_{0}}{\rm{sinc}}({\rm{\Delta }}{\beta }_{mn}{\rm{\Delta }}z / 2)a(t))}^{2}+{d}_{{\rm{\min }}}={d}_{0}{(1+b(t))}^{2}+{d}_{{\rm{\min }}}$$

where *d*_0_ is a constant, and *b*(*t*) is a time variant function related to the external perturbation. The minimum coupling coefficient *d*_min_ is added to represent the minimum inherent mode coupling coefficient. Here, *d*_min_ is time invariant and hence can be neglected. The normalized coupling coefficient *D* is directly proportional to the coupling coefficient *d* as *D* = *δ* ^2^ *d*. Hence the time varying expression *D* = *D*_0_(1 + *b*(*t*))^2^ is expected. Thus, the Equation (5) can be further modified and we then obtain Equation (1).

The Crank Nicolson scheme was used to numerically simulate the power flow redistribution. In summary, by replacing the differential operation by the difference operation in Equation (1), the power flow after the step of propagation can be related to the power flow before the step of propagation through Equation (12), in which u^m^_j_ is the field at *m* ^th^ grid along *z* axis and *j* ^th^ grid at *θ* axis,

12 $${a}_{j}{u}_{j-1}^{m+1}+{b}_{j}{u}_{j}^{m+1}+{c}_{j}{u}_{j+1}^{m+1}={r}_{j}$$

with the coefficients given by,

13 $${a}_{j}=\frac{\alpha {\rm{\Delta }}Z}{2\theta (j){\rm{\Delta }}\theta }-\frac{\alpha {\rm{\Delta }}Z}{{\rm{\Delta }}{\theta }^{2}}$$

14 $${b}_{j}=1+\frac{2\alpha {\rm{\Delta }}Z}{{\rm{\Delta }}{\theta }^{2}}$$

15 $${c}_{j}=-\frac{\alpha {\rm{\Delta }}Z}{2\theta (j){\rm{\Delta }}\theta }-\frac{\alpha {\rm{\Delta }}Z}{{\rm{\Delta }}{\theta }^{2}}$$

16 $$\begin{array}{rcl}{r}_{j} & = & (\frac{(1-\alpha ){\rm{\Delta }}Z}{{\rm{\Delta }}{\theta }^{2}}-\frac{{\rm{\Delta }}Z(1-\alpha )}{2\theta (j){\rm{\Delta }}\theta }){u}_{j-1}^{m}+(1-\frac{2(1-\alpha ){\rm{\Delta }}Z}{{\rm{\Delta }}{\theta }^{2}}){u}_{j}^{m}\\  &  & +(\frac{(1-\alpha ){\rm{\Delta }}Z}{{\rm{\Delta }}{\theta }^{2}}+\frac{{\rm{\Delta }}Z(1-\alpha )}{2\theta (j){\rm{\Delta }}\theta }){u}_{j+1}^{m}\end{array}$$

where *α* is the Crank Nicolson scheme parameter, *θ*(*j*) is the *j* ^th^ point at the mesh grid along *θ* axis, Δ*θ* is the size of mesh grid and Δ*Z* is the propagation step length in *z* axis multiplied by the coupling coefficient *D*. Thomas algorithm was used to solve the linear equations formed by Equation (12).

To obtain Fig. 1, the power flow was first computed. Specifically, the time variable *t* was assigned with 40 evenly spaced values between the span [0, *T*] to generate 40 different coupling coefficients *D* modulated by a sine perturbation, *D* = *D*_0_(1 + *A*_m_sin (2π*t*/*T*))^2^. Then 40 intensity profiles were obtained, 4 of which, i.e. *t* = 0, *t* = 0.25 *T*, *t* = 0.50 *T*, and *t* = 0.75 *T*, were shown in Figs 1(b and c) and 3(a). The power flow projected at the fiber output image plane can be expressed as^6, 8^,

17 $${P}_{T}={\int }_{{\theta }_{low}}^{{\theta }_{up}}sin(\theta )P(\theta )d\theta \approx {\int }_{{\theta }_{low}}^{{\theta }_{up}}\theta P(\theta )d\theta $$

where *θ*_low_ is the lower boundary of the window and *θ*_up_ is the upper boundary of the window. Then, the power detected by the window under the time varying coupling coefficient was calculated and shown in Figs 1(b1,b2,c1 and c2) and 3(b and c).
